# Exploration of carotenoid-producing *Rhodotorula* yeasts from amazonian substrates for sustainable biotechnology applications

**DOI:** 10.1016/j.crmicr.2025.100373

**Published:** 2025-03-08

**Authors:** Raissa Sayumy Kataki Fonseca, Kevyn Melo Lotas, Ana Claudia Alves Cortez, Flávia da Silva Fernandes, Érica Simplício de Souza, Laurent Dufossé, João Vicente Braga de Souza

**Affiliations:** a Programa de Pós-Graduação em Biotecnologia e Recursos Naturais (PPG-MBT) na Universidade do Estado do Amazonas (UEA), Manaus, Amazonas, Brazil; b Instituto Nacional de Pesquisas da Amazônia – INPA. *Av*. André Araújo, 2936 - Petrópolis, 69067-375, Manaus AM, Brazil; c Universidade do Estado do Amazonas – UEA. *Av*. Carvalho Leal, 1777 - Cachoeirinha, 69065-001, Manaus AM, Brazil; d CHEMBIOPRO Lab, Chimie et Biotechnologie des Produits Naturels, ESIROI Agroalimentaire, University of Réunion Island, 97400 Saint-Denis, France

**Keywords:** Pigments, Yeasts, Bioprocess, *Theobroma, Garcinia*, *Euterpe*

## Abstract

Carotenoids are natural pigments responsible for the yellow, orange, and red colors seen in various organisms. The aim of this study was to investigate the potential of yeast strains belonging to the genus *Rhodotorula* isolated from the Amazon Region in the production of carotenoids. Environmental samples from the Amazon Region were collected for yeast isolation. Isolates showing pigmented colonies underwent morphological and biochemical studies, as well as assessment of their potential for carotenoid production. The three best producers were identified through nucleotide sequencing of the ITS1–5.8s-ITS4 rDNA region. The top producer underwent univariate experiments to evaluate the influence of different C/N sources. Carotenoids produced were evaluated using CCD. Results showed the isolation of 13 pigmented yeasts with morphological and biochemical characteristics consistent with the genus *Rhodotorula.* Isolates RGM42 (601 μg/g), RTC42 (362 μg/g), and RTC45 (351 μg/g) stood out as the top carotenoid producers. These yeasts were identified as belonging to the species *Rhodotorula mucilaginosa*. Regarding C/N influence, *R. mucilaginosa* RGM42 produced the highest number of carotenoids using glucose and peptone as carbon and nitrogen sources, respectively. Isolate *R. mucilaginosa* RGM42 exhibited maximum growth with a peak at 72 h of bioprocess. Extracts from isolates *R. mucilaginosa* RGM42, *R. mucilaginosa* RTC42, *and R. mucilaginosa* RTC45 showed retention indices like β-carotene in thin-layer chromatography; however, *R. mucilaginosa* RGM42 presented an additional pigment in its chromatographic profile.

## Graphical abstract

**Figure:**
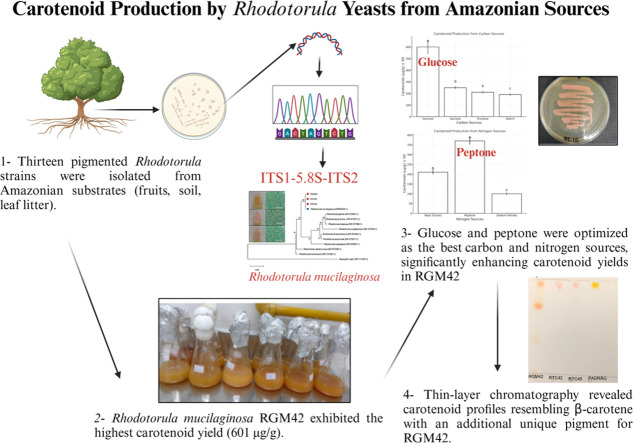

## 1 Introduction

Carotenoids are a diverse group of natural pigments with structural and functional versatility, imparting yellow to red hues across numerous organisms known for their antioxidant and antimicrobial properties, carotenoids find extensive applications in food, pharmaceutical, nutraceutical industries and their applications continue to grow (Choi et al., 2021; Cunha et al., 2023; Ochoa-Viñals et al., 2024).The pharmaceutical, food, and cosmetic industries incorporate carotenoids into nutraceuticals, cosmetics, and supplements due to their medicinal properties. Lutein is used in poultry feed and functional nutrients. Canthaxanthin is applied as a cosmetic additive, in fish feed, and poultry feed. Lycopene is found in nutraceutical cosmetics and dietary supplements, while beta-carotene is used in cosmetics, animal feed, and to enhance meat coloration. Astaxanthin has applications in the cosmetic industry and fish feed, improving the color of the meat (Cardoso et al., 2017). With antioxidant, anti-aging, and anticancer effects, carotenoids are essential in promoting health and well-being(Ashokkumar et al., 2023).

Consumer demand for these compounds has driven a rapidly expanding market, projected to reach $2.7 billion by 2027, with an annual growth rate of 5.7 % (BCC research, 2022). Despite this growing demand, current industrial carotenoids are primarily derived from chemical synthesis or plant sources, both of which present significant limitations. Synthetic carotenoids carry potential toxicity risks, while plant-derived carotenoids are costly and yield-limited (Liu et al., 2021; Ochoa-Viñals et al., 2024). These constraints underline the need for alternative production methods. Biotechnological/Microbial production has emerged as a promising solution, offering advantages such as lower-cost substrates, space-efficient production, and the potential for optimization under controlled conditions. However, challenges remain, including the need for low-cost, sustainable substrates and yield optimization, which must be addressed to make microbial production a viable industrial alternative (Dyaa et al., 2022; Machado et al., 2022; Nguyen et al., 2024; Sharma and Ghoshal, 2020).

Microbial producers of carotenoids, including *Escherichia coli, Gordonia terrae*, and *Rhodotorula* spp., have been extensively studied for industrial applications (Harada, 2021; Loh et al., 2020; Zhao et al., 2019, Zhao et al., 2022). Among these, yeasts such as *Rhodotorula* stand out due to their high carotenoid yields and adaptability to diverse substrates. Despite these advantages, the costs associated with traditional substrates and the limitations of existing processes highlight the need for innovative strategies. Recent studies suggest that agro-industrial residues and other locally available resources could be leveraged as cost-effective substrates (Igreja et al., 2021; Kot et al., 2019; Ochoa-Viñals et al., 2024; Rapoport et al., 2021; Sharma and Ghoshal, 2020). Moreover, while several environments have been explored as reservoirs of carotenoid-producing microorganisms, few studies have investigated tropical ecosystems such as the Amazon rainforest. The Amazon's unique biodiversity, nutrient-rich substrates, and favorable climate conditions make it an ideal environment for microbial diversity, yet research on its pigment-producing fungi remains sparse(Melo Pereira et al., 2024; Oliveira et al., 2022; Sánchez-Muñoz et al., 2020) . Exploring this untapped biodiversity offers an opportunity to identify novel strains with high biotechnological potential.

Despite the Amazon's vast microbial resources, a significant knowledge gap exists regarding its native microorganisms and their potential for industrial carotenoid production. Most research has focused on temperate regions, leaving tropical biomes like the Amazon underrepresented in global studies (Bücker and Bücker-Falcão, 2018; Kimura et al., 2023; Moutinho et al., 2022). By leveraging local microbial resources, it is possible to develop eco-friendly processes that meet the growing global demand for carotenoids while contributing to the conservation of the Amazon rainforest.

This research investigated the carotenoid production potential of *Rhodotorula* yeasts isolated from various Amazonian plant substrates, including fruits, bark, soil, and leaf litter. The main objective was to identify yeast strains with exceptional carotenoid productivity and assess how substrate conditions influenced their yield. By optimizing the selection and characterization of these strains, the study aimed to address one of the major challenges in industrial carotenoid production: high production costs, thus contributing to more sustainable and cost-effective biotechnological processes.

## 2 Material and methods

### 2.1 Sample collection

The sampling strategy included fruit, tree bark, leaves, leaf litter, soil, and three surface water samples, targeting representative Amazonian plant species. The chosen species were *Garcinia macrophylla* Mart., *Theobroma cacao* L., *Pouteria caimito* (Ruiz et Pavon) Radlk., and *Euterpe oleracea* Mart., selected due to their ecological relevance and prevalence in the Amazon rainforest.

To ensure sterile conditions and prevent cross-contamination, all collection tools were autoclaved (121 °C, 15 psi, 20 min, Autoclave Model 600, Phoenix Luferco, Brazil) and wiped with 70 % ethanol between each sample collection. Approximately 20 g of fruit, bark, and leaves were collected from each plant, with each sample individually placed in sterile polyethylene bags to maintain microbial integrity. Leaf litter samples, consisting of freshly fallen leaves around the base of each plant, were also collected and handled similarly.

Soil samples were taken from a depth of 1–3 cm to focus on surface microbial communities. Approximately 50 g of soil per sample were collected and stored in sterile, screw-capped polypropylene containers (Nalgene™, Thermo Fisher Scientific, USA), with each container labeled by sample type, location, and date.

Three surface water samples (500 mL each) were collected from nearby streams within the sampling sites. Water was sampled using pre-sterilized glass bottles (Schott DURAN®, DWK Life Sciences, Germany) to avoid chemical interactions. All collected samples were immediately coded by type, specific collection site, and date to ensure systematic tracking.

Sampling was conducted at the Instituto Nacional de Pesquisas da Amazônia (INPA) – 'Bosque da Ciência' (Latitude South: 3°09′39″, Longitude West: 59°98′77″) in Manaus, Amazonas, Brazil. This site, a forest of primary and secondary vegetation, was selected due to its rich biodiversity. After collection, samples were stored at 4 °C in a portable refrigerator (Coleman® PowerChill™, USA) for transport to the laboratory and processed within 24 h to preserve microbial viability and community diversity.

### 2.2 Isolation of microorganisms

Fruit samples were initially rinsed with distilled water to remove surface impurities, followed by treatment with a 2 % (w/v) sodium hypochlorite solution (Sigma-Aldrich, USA) to ensure surface sterilization. This disinfection step was conducted by immersing the samples in sodium hypochlorite a 2 % (w/v) for 1 min, then washing them three times with sterile distilled water to eliminate any residual disinfectant. Samples were then allowed to air dry at room temperature (25 ± 2 °C) in a laminar flow cabinet Modelo A2 da linha BIOSAFE (Veco, Campinas, Brasil) to prevent contamination. For direct sample processing, other non-fruit materials, such as soil and water, were used without the need for further surface sterilization.

One gram of each sample was weighed on an analytical balance (model AX124, Sartorius, Germany) and transferred to a sterile 15 mL test tube containing 9 mL of autoclaved distilled water, achieving a 10-fold dilution. The mixture was vortexed (15 s at 3000 rpm) in a vortex mixer (model Vortex-Genie 2, Scientific Industries, USA) to homogenize the sample. Serial dilutions were then prepared up to 10⁻⁴ by transferring 1 mL from each dilution into a subsequent tube containing 9 mL of sterile distilled water. From each dilution, 100 μL was plated onto Petri dishes containing YEPD medium (1 % yeast extract, 2 % peptone, 2 % glucose, 2 % agar, and 250 mg/L chloramphenicol) to selectively support yeast growth and inhibit bacterial contaminants (Indunil Chinthani et al., 2020; Oliveira et al., 2024).

The 100 μL aliquots were spread uniformly across the medium surface using a sterilized Drigalsky loop, ensuring even distribution of microbial cells. Each inoculation was performed in triplicate to ensure reproducibility, and plates were incubated at 28 °C in an incubator (BOD, Quimis, Brazil) for 120 h. After incubation, plates were inspected for colonies exhibiting distinct pigmentation, ranging from yellow to red. These colonies, indicative of potential carotenoid-producing yeasts, were subcultured onto fresh YEPD plates to isolate and confirm colony purity. Colony morphology, including pigmentation, was observed to assist in preliminary identification of yeast isolates.

### 2.3 Identification of yeasts

Yeasts exhibiting visible pigmentation were initially subjected to detailed morphological and biochemical characterization. Isolates that demonstrated high carotenoid production were subsequently selected for molecular identification to confirm their taxonomic classification and to allow comparative phylogenetic analysis.

#### 2.3.1 Morphological identification

For morphological identification, pigmented yeast isolates were streaked using the exhaustion method on Petri dishes containing YEPD (Yeast Extract-Peptone-Dextrose) medium. The medium was prepared by adding 1 % yeast extract, 2 % peptone, 2 % glucose, and 2 % agar, and plates were autoclaved at 121 °C for 15 min. The inoculated plates were then incubated at 30 °C for 120 h in a controlled incubator (Thermo Scientific™ Heratherm™, Germany). Colony morphology was assessed based on specific traits such as size, shape, elevation, edge type, surface structure, color, and sheen under a stereomicroscope (Olympus SZ61, Olympus Corporation, Japan). The colony characteristics were compared with standard morphological descriptions to aid in preliminary classification at the genus level. Observations were repeated in triplicate to confirm consistency across samples (Fernandes et al., 2024).

#### 2.3.2 Biochemical identification

Biochemical identification was performed by assessing the ability of each isolate to assimilate and ferment different carbon sources. For the assimilation tests, isolates were first cultured on YEPD medium, incubated for 120 h at 28 °C, then transferred to tubes containing 5 mL of autoclaved distilled water. Cell suspensions were standardized at 1 × 10⁶ cells/mL using a Neubauer chamber (Boeco, Germany). Two milliliters of the standardized suspension were seeded on Petri dishes containing Nitrogen Base Medium (Difco™, BD Biosciences, USA) supplemented with 2 % agar to create a solid auxanogram medium. Test carbohydrates, including sucrose, galactose, lactose, maltose, fructose, glucose, and raffinose, were prepared in the form of filter discs (5 mm diameter) impregnated with 10 µL of each carbohydrate solution (at a concentration of 0.5 M) and distributed equidistantly on the medium. Plates were incubated at 28 °C for 72 h, and results were observed by noting turbidity halos indicating carbon source assimilation.

For the fermentation tests, isolates were inoculated in test tubes containing 10 mL of Nitrogen Base Medium (Difco™, BD Biosciences, USA) supplemented with 0.5 % of the selected carbon sources (sucrose, glucose, maltose, and lactose). Durham tubes were placed in each test tube to capture any gas produced during fermentation. The tubes were autoclaved at 120 °C for 15 min before inoculation. After inoculating with 0.5 mL of each isolate suspension, the tubes were left at room temperature for a maximum period of 30 days. Positive results were confirmed by observing gas bubbles within the Durham tube, indicating active fermentation(Lacaz et al., 2002; Reiss et al., 2011).

#### 2.3.3 DNA sequencing identification

Among the isolated strains, the three top carotenoid producers with morphological features consistent with the genus *Rhodotorula* were selected for molecular identification. DNA extraction was performed according to the protocol described by (Sambrook et al., 1989) using a phenol-chloroform-isoamyl alcohol extraction method. Yeast cells were collected from YEPD agar plates, resuspended in 500 µL of lysis buffer (50 mM Tris–HCl, 10 mM EDTA, 1 % SDS), and homogenized using a vortex mixer. Following extraction, DNA purity and concentration were verified using a Basic 5 (Eppendorf, Hamburg, Germany).

For amplification of the ITS region, the primer pairs ITS1 (5′-TCCGTAGGTGAACCTGCGG-3′) and ITS4 (5′-TCCTCCGCTTATTGATATGC-3′) were used (White et al., 1990). PCR reactions were carried out in a final volume of 25 µL, containing 2.5 µL of 10X PCR buffer, 1.5 mM MgCl₂, 0.2 mM dNTPs, 0.2 µM of each primer, 1.25 U of Taq DNA Polymerase (Thermo Scientific, USA), and 1 µL of template DNA (approximately 50 ng/µL) using a Veriti 96-well thermal cycler (Applied Biosystems, Thermo Fisher Scientific, Waltham, MA, USA). Amplification conditions were as follows: an initial denaturation at 95 °C for 5 min; 35 cycles of denaturation at 95 °C for 1 min, annealing at 53 °C for 1 min, and extension at 72 °C for 1 min; with a final extension at 72 °C for 7 min. PCR products were analyzed on 1 % agarose gel electrophoresis (Bio-Rad, USA) stained with ethidium bromide (0.5 µg/mL) and visualized under UV light.

The resulting PCR products were purified using the BigDye Terminator v3.1 kit (Applied Biosystems, ThermoFisher, USA) following the manufacturer's protocol. Purified products were precipitated with ethanol/EDTA, and nucleotide sequencing was performed using an ABI PRISM 3130xL automated sequencer (ThermoFisher, USA). Sequences were edited and aligned using the BioEdit software (version 7.0.9.0), and then deposited in the GenBank database (accession numbers available in Supplementary Material 1). BLASTN analysis was conducted to compare the sequences against the NCBI database, considering a similarity threshold above 97 %.

Phylogenetic analysis was carried out using the MEGA X software, with sequences from closely related species obtained from NCBI. Genetic distance analysis was performed using the Neighbor-Joining (NJ) method, with 1000 bootstrap replicates to validate the clusters. These analyses confirmed the identity and phylogenetic relationships of the yeast isolates, particularly those with significant carotenoid production capabilities.

### 2.4 Rhodotorula screening for carotenoid production

To initiate the carotenoid production process, a loopful of *Rhodotorula* spp. cells was collected aseptically and suspended in 10 mL of autoclaved distilled water within a 15 mL sterile Falcon™ tube (Corning, USA). The suspension was vortexed briefly to ensure homogeneity, and the cell density was adjusted to 1 × 10⁷ cells/mL, verified using a Neubauer chamber (Brand™, Germany) to standardize cell concentration.

The bioprocess protocol was adapted from (Dyaa et al., 2022) to optimize the yield of carotenoid pigments. The initial culture medium was prepared by transferring of the pre-inoculum suspension 1 × 105/mL to 50 mL of YEPD broth (1 % yeast extract, 2 % peptone, and 2 % glucose) in a 125 mL Erlenmeyer flask (Duran™, Germany). Each flask was covered with a sterile cotton plug and placed on an orbital shaker (IKA KS 260 basic, IKA Works, Germany) set to 100 rpm to maintain aerobic conditions. Cultures were incubated at 28 °C for 120 h under continuous agitation to ensure adequate nutrient distribution and oxygen transfer. Experiments were performed in triplicate to ensure reproducibility and statistical reliability.

Following incubation, a 10 mL aliquot of each culture broth was transferred to a 15 mL Falcon™ tube (Corning, USA) and centrifuged at 5500 x g for 20 min at 25 °C using an Eppendorf 5810R centrifuge (Eppendorf AG, Germany) to separate the cells from the supernatant. After centrifugation, the supernatant was carefully decanted, and 1 mL of dimethyl sulfoxide (DMSO) (Sigma-Aldrich™, USA) was added to the cell pellet along with sterile glass beads (0.5 mm diameter, BioSpec Products, USA) to facilitate cell lysis. The cell suspension was vortexed (Vortex-Genie 2, Scientific Industries, USA) for 1 min and incubated at 55 °C for 1 hour, with intermittent vortexing every 15 min to enhance carotenoid extraction from the yeast cell walls.

Subsequent extraction steps involved the addition of 2 mL of a solvent mixture composed of acetone and petroleum ether in a 1:1 (v/v) ratio. The mixture was vortexed briefly and centrifuged at 5500 x g for 15 min under the same conditions. This process resulted in phase separation, with the carotenoid-rich upper petroleum ether phase showing characteristic orange or pink coloration, indicating the presence of carotenoids. This upper layer was carefully pipetted into a separate container and stored in amber vials to prevent photooxidation. The extraction procedure was repeated four times to maximize the recovery of carotenoids. All extraction steps were performed under minimal light exposure to avoid pigment degradation, ensuring the integrity of the carotenoid compounds.

Each extracted phase was subsequently subjected to quantitative analysis, with total carotenoid concentration. Bioprocess samples were also collected to quantify yeasts biomass.

### 2.5 Analytical methods

#### 2.5.1 Determination of total carotenoids

The quantification of total carotenoids was conducted using a spectrophotometer (BioSpectrometer® basic, Eppendorf, Germany) set at a wavelength of 450 nm, appropriate for detecting β-carotene as the primary carotenoid(Kreft and Kreft, 2010). Carotenoid extracts were prepared by suspending the biomass in a mixture of solvents, ensuring complete extraction of pigments. Petroleum ether was used as a blank reference in the spectrophotometric readings, a common practice due to its compatibility with carotenoid solubility. Prior to analysis, the spectrophotometer was calibrated to eliminate background absorbance interference, providing an accurate baseline for carotenoid quantification.

Eq. (1):

(1) $$TC=\frac{A\;x\;V\;x\;10^{6}}{A^{1}\left(1cm\right)\;x\;100x\;m\left(sample\right)}$$

• TC represents the total carotenoid concentration (μg/g),
• A denotes the absorbance obtained at 450 nm,
• V is the total volume of solvent (mL) used for resuspension,
• m_sample represents the sample mass (g),
• A_{1cm}^1 is the specific absorption coefficient of β-carotene in petroleum ether, standardized at 2592 L·g⁻¹·cm⁻¹​.

The samples were processed in triplicate to ensure accuracy and reproducibility. Upon reading the absorbance values, carotenoid concentrations were calculated and recorded, expressing the results in μg of carotenoids per g of dry biomass (μg/g).

#### 2.5.2 Determination of final dry biomass

The final dry biomass (P_biomass) was determined by measuring the dry weight of homogenized cultures (Rice et al., 2017). A volume of 10 mL from each culture broth was filtered using pre-weighed filter paper and placed in a pre-dried Petri dish. These Petri dishes were previously dried at 105°C for 24 h to ensure complete removal of moisture and establish a consistent baseline weight.

After the initial weight (Pi) of the dish and filter paper was recorded, samples were oven-dried at 105°C until achieving constant weight, a process that typically required 24 h. After drying, the final weight (Pf) was recorded. The dry biomass was calculated using the equation below:

Eq. (2):

(2) P(biomassa)=Pi−Pf

where:

• Pi is the initial weight of the dish and filter paper before drying,
• Pf is the final weight post-drying.

The results were expressed in grams per liter (g/L), providing a standardized measure of biomass yield. Each determination was performed in triplicate to account for any procedural variability and ensure statistical significance.

#### 2.5.3 Thin layer chromatography

Thin layer chromatography (TLC) was employed to analyze and separate the carotenoid compounds present in the extracts(Waksmundzka-Hajnos and Sherma, 2011). This technique was chosen for its effectiveness in providing a rapid and semi-quantitative assessment of carotenoid composition. A β-carotene standard solution, prepared at a concentration of 1.0 mg/mL and diluted in dichloromethane, was used as a reference. The carotenoid extracts from the three highest-yielding strains were also diluted in petroleum ether to match the concentration of the standard, allowing for direct comparison.

Each sample was applied as a spot on a TLC plate (silica gel, 60 F₂₅₄, Merck, Germany) at equal distances (0.5 cm from the base), ensuring uniform application and enabling consistent elution. The plate was then developed in a mobile phase consisting of ethyl acetate/formic acid/water in a 6:1:1 (v/v) ratio. This eluent system was selected for its compatibility with the polarity of carotenoids, facilitating effective migration across the plate surface.

Following development, the retention factor (Rf) for each carotenoid spot was calculated using the equation:

Eq. (3):

(3) $$Rf=\frac{dc}{ds}$$

where:

• Rf is the retention factor, a dimensionless value representing compound migration,
• d_c is the distance traveled by the carotenoid compound,
• d_s is the distance traveled by the solvent front.

Rf values were compared with those of the standard to identify carotenoid types and assess the purity of the extracts. The experiment was performed in triplicate to ensure the consistency of results and facilitate reproducibility.

### 2.6 Statistical analysis

Data was statistically analyzed to determine mean values and standard deviations using Excel Software (Microsoft Corporation®, Redmond, WA, USA). For comparisons between groups, analysis of variance (ANOVA) was applied to assess the significance of observed differences in measured variables. The normality of the data distribution was confirmed using the Shapiro-Wilk test prior to performing the ANOVA. Post hoc comparisons were conducted using Tukey's Test to evaluate pairwise differences among means, applying a confidence level of 95 % to ensure statistical rigor and accuracy in the interpretation of results.

To enhance the reliability of the statistical interpretations, additional software, including MINITAB 17 (Minitab, LLC, State College, PA, USA), was employed for ANOVA and Tukey's post hoc analyses, enabling precise evaluation of treatment effects. For all analyses, significance was defined at *p*< 0.05, with highly significant differences reported at *p*< 0.01 where relevant. Data processing and visualization were also assisted by R software (R Foundation for Statistical Computing, Vienna, Austria), particularly for creating box plots, histograms, and residual plots to illustrate distribution and variance patterns.

For data integrity, BioEdit software (Ibis Biosciences®, Carlsbad, CA, USA) and MEGA 10 (Molecular Evolutionary Genetics Analysis, Temple University, Philadelphia, PA, USA) were utilized in phylogenetic analyses where applicable (Hall, 1999; Kumar et al., 2018).

## 3 Results

### 3.1 Isolation of microorganisms

Environmental samples collected from Amazonian fruit trees were investigated to determine the prevalence and distribution of yeasts belonging to the genus *Rhodotorula*. Table 1 illustrates the isolation frequencies from various substrates including fruits, tree bark, leaf litter, soil, and water from the *Bosque* da *Ciência* - Instituto Nacional de Pesquisas da Amazônia (INPA), Manaus, Brazil. The yeast colonies morphologically compatible with *Rhodotorula* were quantified as colony-forming units per gram (CFU/g) of sampled material. Notably, leaf litter and fruits exhibited varying CFU counts, with the highest occurrence observed in *Garcinia macrophylla Mart.* leaf litter (4.9 × 10⁴ total fungi CFU/g, 3 × 10³ *Rhodotorula* CFU/g) and *Theobroma cacao* L. fruits (1.4 × 10⁴ total fungi CFU/g, 4 × 10³ *Rhodotorula* CFU/g).

**Table 1:** Number of CFU/g of total fungi and colonies morphologically compatible with the genus *Rhodotorula* isolated from environmental samples collected at from Amazonian Fruit Trees from Instituto Nacional de Pesquisas da Amazônia.

Source of Isolation	Collected Part	Total Fungi (CFU/g)	*Rhodotorula* number (CFU/g)	Isolated Yeast Codes
*Garcinia macrophylla* Mart.	Fruit	1.4 × 10³	0	
	Tree Bark	3.2 × 10³	0	
	Leaf Litter	4.9 × 10⁴	3 (3 × 10³)	RMG41 RGM42 RGM43
	Soil	8.2 × 10⁴	0	
*Theobroma cacao* L.	Fruit	1.4 × 10⁴	4 (4 × 10³)	RTC41RTC42 RTC43 RTC44
	Tree Bark	1.2 × 10⁴	2 (2 × 10³)	RTC45 RTC46
	Leaf Litter	3.2 × 10⁴	0	
	Soil	4.3 × 10⁴	0	
*Pouteria caimito* (Ruiz et Pavon) Radlk.	Fruit	2.3 × 10³	2 (2 × 10^2^)	RPC41 RPC42
	Tree Bark	1.2 × 10³	0	
	Leaf Litter	2.5 × 10⁴	0	
	Soil	3.3 × 10³	0	
*Euterpe oleracea* Mart.	Fruit	2.9 × 10²	0	
	Tree Bark	5.6 × 10³	0	
	Leaf Litter	5.6 × 10³	0	
	Soil	2.2 × 10⁴	1 (1 × 10^3^)	REO41
Water - Bosque da Ciência	Sample 1	4 × 10³	0	
	Sample 2	2 × 10⁴	1(1 × 10^3^)	RAB41
	Sample 3	2.1 × 10⁴	0	

### 3.2 Carotenoid production by Rhodotorula strains

Investigation into carotenoid production by different strains of *Rhodotorula* revealed significant variations across the isolates, as shown in Table 2. The study, conducted over 120 h of fermentation in YEPD medium, focused primarily on the yield of total carotenoids relative to biomass production. Among the strains, *Rhodotorula* RGM42 was identified as the most prolific producer, yielding 601 ± 52 μg/g of carotenoids, which was statistically higher (*p*< 0.05, ANOVA followed by Tukey's test) than the yields observed in other strains. Conversely, the strains *Rhodotorula* RTC46 and *Rhodotorula* REO41 demonstrated markedly lower carotenoid yields of 48.45 ± 1.93 μg/g and 41.70 ± 15.23 μg/g, respectively.

**Table 2:** Biomass and total carotenoids produced in 120 h of bioprocess in YEPD medium by *Rhodotorula* strains from environmental samples collected at the Instituto Nacional de Pesquisas da Amazônia.

Code	Biomass (g/L) *	Total Carotenoid (μg/g)
***Rhodotorula* RGM42**	**6.7 ± 0.1**	**601.0± 52.0^a^**
***Rhodotorula* RTC42**	6.8 ± 0.2	362.0 ± 21.0^b^
***Rhodotorula* RTC45**	6.42 ± 0.5	351.0 ± 11.0^b^
***Rhodotorula* RGM43**	7.0 ± 0.1	161.0 ± 23.0^c^
***Rhodotorula* RAB41**	6.2 ± 0.4	114.0 ± 44.0^c, d^
***Rhodotorula* RGM41**	6.2 ± 0.3	113.0 ± 25.0 ^c, d^
***Rhodotorula* RTC46**	7.00 ± 0.70	48.45± 1.93^d, e^
***Rhodotorula* REO41**	6.50 ± 0.43	41.70 ± 15.23^d, e^
***Rhodotorula* RTC41**	6.84 ± 0.22	34.36 ± 1.06^d, e^
***Rhodotorula* RTC44**	6.68 ± 0.29	31.98 ± 9.62^d, e^
***Rhodotorula* RPC42**	6.36 ± 1.25	21.86 ± 2.99^e^
***Rhodotorula* RTC43**	7.14 ± 0.66	15.56 ± 4.29^e^
***Rhodotorula* RPC41**	7.10 ± 0.38	12.10 ± 1.85^e^

⁎ biomass showed no significant difference at a 95 % confidence level. Different letters in the same column represent a significant statistical difference.

### 3.3 Molecular identification of Rhodotorula strains

**Fig. 1:**
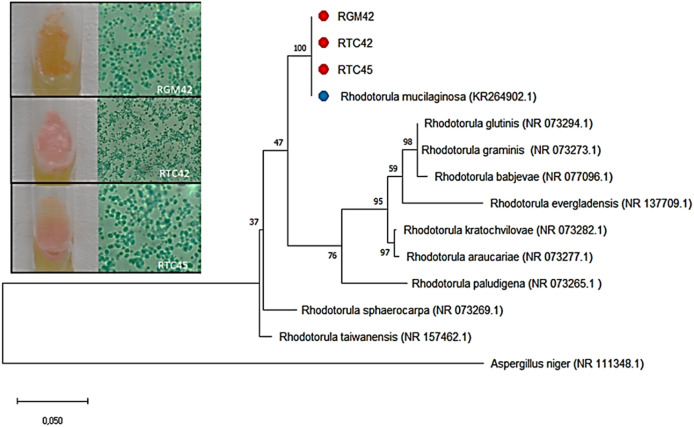
Biomolecular identification of yeasts isolated from environmental samples collected at from amazonian fruit trees from Instituto Nacional de Pesquisas da Amazônia. Phylogenetic tree constructed using ITS1–5.8S-ITS4 region sequencing. Accession numbers are labeled on the sequences. Support values are from Bayesian inference and maximum likelihood analyses performed in MEGA X. Red dots represent isolates from this study, while blue dots indicate major species with similar sequencing.

To ascertain which *Rhodotorula* species are the most prolific carotenoid producers, DNA sequencing identification was conducted, as depicted in Fig. 1. This figure presents the phylogenetic relationships of yeast strains isolated from environmental samples taken from Amazonian fruit trees at the Instituto Nacional de Pesquisas da Amazônia. The isolates RGM42, RTC42, and RTC45 were subjected to sequencing of the ITS1–5.8S-ITS4 region and compared against sequences in the GenBank database. The comparison revealed that all three isolates exhibited a 99 % similarity to the reference sequence *R. mucilaginosa* KR264902.1. Based on morphological, biochemical, and molecular characteristics, the yeasts were classified as *R. mucilaginosa* RGM42, RTC42, and RTC45, respectively.

### 3.4 Effect of carbon and nitrogen sources on carotenoid production

**Fig. 2:**
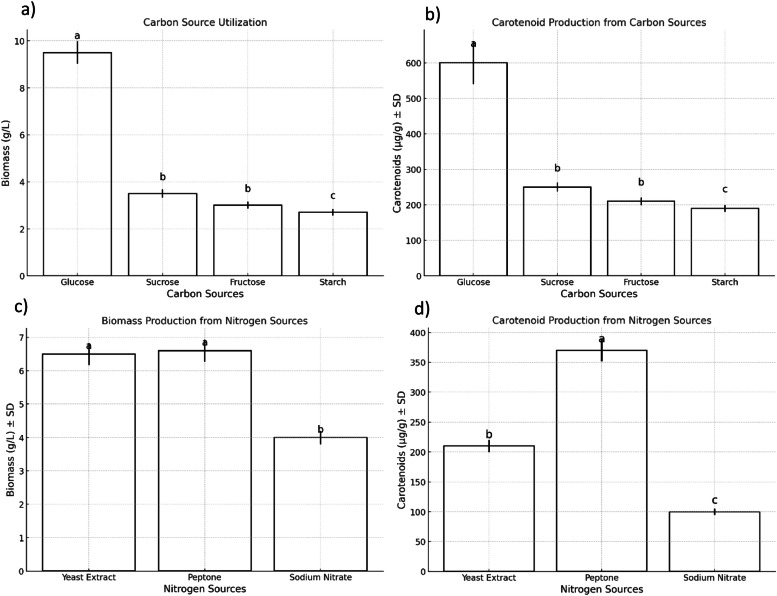
Comparative Analysis of Different Carbon and Nitrogen Sources on Biomass and Carotenoid Production by *Rhodotorula mucilaginosa* RGM42. Statistical significance was assessed using Student's *t*-tests for each source compared against all others within its group (carbon or nitrogen). Sources that did not significantly differ from each other in terms of biomass or carotenoid production are marked with the same letter (a, b, c, etc.), with the letters assigned based on ascending mean values.

Investigating the optimal carbon and nitrogen sources for carotenoid production by the isolate *R. mucilaginosa* RGM42, distinct carbon sources were assessed for their influence on biomass accumulation and carotenoid synthesis. The experiments demonstrated significant variability among the carbon sources tested. Glucose emerged as the superior carbon source, facilitating the highest biomass production of 9.5 ± 0.475 g/L and carotenoid content of 600 ± 60 μg/g, as depicted in Fig. 2.

The influence of various nitrogen sources on the production of carotenoids by *R. mucilaginosa* RGM42 was also explored. Among the nitrogen sources evaluated, peptone was found to be the most effective, supporting a biomass of 6.6 ± 0.33 g/L and a carotenoid concentration of 370 ± 18.5 μg/g (Fig. 2).

### 3.5 Carotenoid analysis by thin layer chromatography (TLC)

**Fig. 3:**
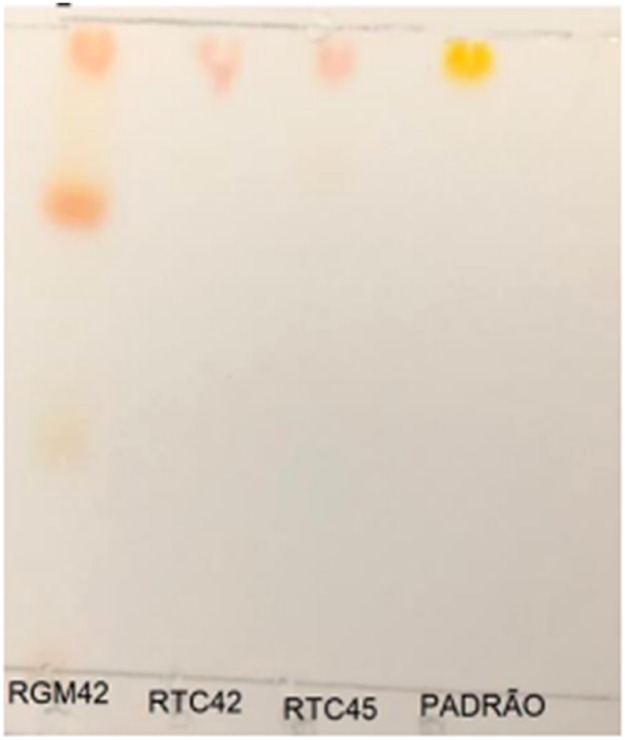
Thin-layer chromatography of extracts containing carotenoids from isolates *R. mucilaginosa* RGM42, *R. mucilaginosa* RTC42 and *R. mucilaginosa* RTC45, using beta-carotene as a standard. Thin layer chromatography using acetic acid: formic acid: water in the proportion 6:1:1 v/v as a system. Standard: β-carotene.

Thin-layer chromatography (TLC) was employed to investigate the components present in the extracts of the top three producers. As shown in Fig. 3, the extract of *R. mucilaginosa* RGM42 revealed two distinct bands. Additionally, the extracts of *R. mucilaginosa* RGM42, RTC42, and RTC45 exhibited a band with a chromatographic profile similar to that of the carotenoid β-carotene. These profiles indicate the potential diversity of carotenoids produced by these strains, warranting further structural elucidation.

## 4 Discussion

This study provides an analysis of the prevalence of *Rhodotorula* species in various substrates from Amazonian fruit trees and highlights their capacity for carotenoid biosynthesis, with a focus on *Rhodotorula mucilaginosa* RGM42.

Among the samples, *Garcinia macrophylla Mart.* leaf litter and *Theobroma cacao* L. fruits demonstrated the highest concentrations of *Rhodotorula*, yielding 3 × 10³ CFU/g and 4 × 10³ CFU/g, respectively. The occurrence of *Rhodotorula* in diverse substrates aligns with its well-documented adaptability and global distribution across environments, including soil, water, and plant material due to their metabolic versatility and ability to dominate microenvironments rich in organic matter (Ochoa-Viñals et al., 2024; Zhao et al., 2022). This research pioneers the quantitative description of *Rhodotorula* in Amazonian substrates.

The carotenoid yield obtained by strain RGM42 (601 ± 52 μg/g) is consistent with previously reported values for similar genera (Dyaa et al., 2022; Ghilardi et al., 2020; C U Mussagy et al., 2021). For instance, *Rhodotorula gracilis* ATCC 10,788 cultivated in bioreactors produced approximately 150 μg/g (Kot et al., 2019), while the same strain grown in potato wastewater yielded only 6.24 μg/g (Kot et al., 2020). Similarly, *R. mucilaginosa* MK1 (LC527461.1) and *R. mucilaginosa* CMIFS 004, isolated from birch forests in Poland, both yielded approximately 346 μg/g. Meanwhile, *R. glutinis* CCT-2186 reached up to 450 μg/g under optimized conditions (Mussagy et al., 2021). These comparisons highlight the industrial potential of RGM42, particularly as it achieved high yields without extensive optimization. The superior yield of RGM42 compared to the other isolates from the present work (RTC42 and RTC45) may result from an adaptive advantage, as carotenoids play essential roles in oxidative stress protection, UV radiation defense, and energy storage (Frengova and Beshkova, 2009). Differences in carotenoid biosynthesis among *Rhodotorula* strains are likely influenced by genetic variation, metabolic regulation, and environmental pressures, which can shape pigment production capabilities. While these results are promising, *Rhodotorula* species still lag behind *Blakeslea trispora* in industrial carotenoid yield, reaching only 30–40 mg/g dry weight under optimized conditions (Mehta et al., 2003; Sandmann, 2022). Advances in genetic engineering, such as crtYB overexpression in *R. glutinis*, have demonstrated the potential to enhance production, achieving β-carotene yields of 27.1 mg/g (Pi et al., 2018). These findings underscore the need for further optimization of Amazonian strains to enhance their industrial competitiveness.

Molecular identification using ITS sequencing confirmed that isolates RGM42, RTC42, and RTC45 shared 99 % similarity with *R. mucilaginosa*, validating their classification within this species. *Rhodotorula mucilaginosa* is recognized for its biotechnological relevance, being used in pigment production and other applications (Ghilardi et al., 2020). Studies have highlighted its versatility in producing valuable compounds, including carotenoids, lipids, and enzymes,making it an attractive candidate for industrial exploitation. These findings further support the relevance of investigating Amazonian isolates of *R. mucilaginosa* as a microbial resource with untapped potential.

Carotenoid extracts from RGM42, RTC42, and RTC45 were characterized using thin-layer chromatography (TLC), revealing two prominent TLC bands in the RGM42 extract. This genus is known to produce carotenoids such as β-carotene, torulene, and torularhodin, all of which have significant industrial and nutritional applications. β-Carotene is widely used as a natural pigment and antioxidant, serving as a precursor for vitamin A. Torulene and torularhodin, on the other hand, are valuable for their antioxidant and potential anticancer properties, with applications in pharmaceuticals and nutraceuticals. The biosynthesis of these carotenoids in *Rhodotorula mucilaginosa* follows the isoprenoid pathway, starting with the condensation of isopentenyl pyrophosphate (IPP) and dimethylallyl pyrophosphate (DMAPP) to form geranylgeranyl pyrophosphate (GGPP). This reaction is catalyzed by GGPP synthase (crtE). GGPP is then converted into phytoene by phytoene synthase (crtYB), which is the first committed step in the pathway. Phytoene undergoes sequential desaturation reactions catalyzed by phytoene desaturase (crtI), leading to the formation of lycopene. In Rhodotorula, lycopene is further modified by lycopene cyclase (crtYB) to produce β-carotene. Additionally, torulene and torularhodin are synthesized through the action of specific desaturases and hydroxylases (Tang et al., 2019), conferring distinct structural modifications that enhance their bioactive properties.

The optimization of carbon and nitrogen sources significantly influenced carotenoid production by RGM42. Among the tested carbon sources, **glucose** supported the highest biomass yield (9.5 ± 0.475 g/L) and carotenoid concentration (600 ± 60 μg/g). This aligns with the well-documented preference of *Rhodotorula* spp. for fermentable sugars, which are directly metabolized via the glycolytic pathway, providing precursors such as acetyl-CoA for carotenoid biosynthesis. **Sucrose**, as a disaccharide, requires hydrolysis into glucose and fructose before entering glycolysis, which may introduce an additional metabolic step and slightly lower carotenoid yields. In contrast, **fructose** and **starch** necessitate additional enzymatic conversions before they can be efficiently utilized, potentially leading to lower metabolic flux toward carotenoid biosynthesis. Fructose, despite being a monosaccharide, enters glycolysis via fructokinase and bypasses the initial regulatory steps controlled by hexokinase and phosphofructokinase, possibly altering metabolic regulation. Starch, a polysaccharide, requires extracellular hydrolysis into simpler sugars before cellular uptake, adding complexity to its utilization and possibly limiting its efficiency as a sole carbon source. Similarly, the choice of nitrogen source affected pigment synthesis. **Peptone** proved to be the most effective, yielding a carotenoid concentration of 370 ± 18.5 μg/g and biomass of 6.6 ± 0.33 g/L. Peptone provides a broad spectrum of amino acids, which may enhance flux through the mevalonate pathway, a key route for isoprenoid biosynthesis and carotenoid accumulation (Kot et al., 2019). In contrast, inorganic nitrogen sources such as **ammonium** and **nitrates** may limit metabolic flexibility, leading to suboptimal pigment production. To enhance the economic feasibility of microbial carotenoid production, future studies should explore the replacement of glucose and peptone with low-cost, sustainable substrates, such as agro-industrial residues, which could serve as alternative carbon and nitrogen sources while reducing production costs and environmental impact.

This study has some limitations that should be addressed in future research. The use of sterile distilled water for dilutions, instead of physiological fluids, may not fully represent real biological conditions. Additionally, while TLC provided preliminary insights into carotenoid production, more advanced analytical techniques such as HPLC are required for precise identification and quantification, allowing a more accurate assessment of the biosynthetic potential of *Rhodotorula* strains. Another important limitation is the lack of industrial-scale evaluation, which is essential to determine the feasibility of large-scale biotechnological applications. Future studies should focus on optimizing culture conditions—such as carbon and nitrogen sources, aeration, and pH control—to enhance carotenoid production. Moreover, scaling up fermentation processes and assessing economic viability under industrial conditions will be crucial to validate the potential of these strains for sustainable pigment production.

The findings of this study offer a novel perspective on the biotechnological potential of Amazonian yeasts, particularly *Rhodotorula mucilaginosa* RGM42. By identifying strains with high carotenoid yields and exploring their metabolic pathways, this research provides a foundation for developing sustainable processes for natural pigment production. Carotenoids are well-known for their antioxidant properties, which contribute to skin health, immune system support, and the prevention of oxidative stress-related diseases. Given these bioactive properties, the pigments produced by *R. mucilaginosa* hold promise for applications in the food industry as natural colorants and functional ingredients, in the pharmaceutical sector as potential supplements with antioxidant benefits, and in cosmetics for skin protection and anti-aging formulations. Furthermore, leveraging agro-industrial residues as substrates could align these processes with the principles of the bioeconomy, promoting sustainability and reducing production costs. The creation of a global or Amazon-specific collection of *Rhodotorula* strains is a potential strategy to enhance research on carotenoid biosynthesis, enabling large-scale production and bioindustrial innovation. This initiative would also highlight the Amazon's role as a reservoir of microbial diversity with significant contributions to global sustainability efforts.

## 5 Conclusion

This study identifies *Rhodotorula mucilaginosa* strains from Amazonian substrates as new promising carotenoid producer, with **RGM42** yielding 601 µg/g under optimized conditions. Further exploration of these strains can enhance their industrial viability while supporting the conservation and sustainable use of Amazonian biodiversity.

## Funding

We gratefully acknowledge the financial support from FAPEAM (Fundação de Amparo à Pesquisa do Estado do Amazonas), CNPq (Conselho Nacional de Desenvolvimento Científico e Tecnológico), and CAPES (Coordenação de Aperfeiçoamento de Pessoal de Nível Superior), under process number 062.00898/2019. The project, titled “Production, Stability, and Applicability of Colorants Produced by Filamentous Fungi Isolated from Soil Samples of the Amazon Region”, was funded by the Call No 006/2019 - UNIVERSAL AMAZONAS. The project was coordinated by João Vicente Braga de Souza.

## Editorial declaration

This publication in Current Research in Biotechnology is the only valid version of this article and has been approved by the author.

## Declaration of competing interest

The authors declare that they have no known competing financial interests or personal relationships that could have appeared to influence the work reported in this paper.

## Data Availability

Data will be made available on request.

## Appendix Supplementary materials

Image, application 1
