# Supplementary material for: Exploration of carotenoid-producing Rhodotorula yeasts from amazonian substrates for sustainable biotechnology applications
Source: Curr Res Microb Sci. 2025 Mar 8;8:100373. doi: 10.1016/j.crmicr.2025.100373 (PMC11964568; doi:10.1016/j.crmicr.2025.100373)
Supplement: Supplementary file 1 [file mmc1.docx]

# Supplementary Material 1

**Table 1** Accession Numbers of the Sequences Deposited in the NCBI Database (<https://www.ncbi.nlm.nih.gov/>) for the Species Evaluated in This Study.

| **Isolate code** | **Species** | **NCBI number (ITS)** |
| --- | --- | --- |
| **RGM42** | *Rhodotorula mucilaginosa* | PV257636 |
| **RTC42** | *Rhodotorula mucilaginosa* | PV257637 |
| **RTC45** | *Rhodotorula mucilaginosa* | PV257638 |
